# Myricetin Suppresses Inflammatory Th17 Polarization to Mitigate Alzheimer's Disease Pathogenesis

**DOI:** 10.1111/cns.70644

**Published:** 2025-11-04

**Authors:** Yufei Li, Ao Sun, Jingjing Han, Rui Hong, Cong Cao, Aihua Zhou, Zhengxiang Fan, Linlin Zhang, Xuebin Qu

**Affiliations:** ^1^ Department of Basic Medical Science Jiangsu Medical College Yancheng Jiangsu China; ^2^ Department of Pharmacology, Yancheng First Hospital Affiliated Hospital of Nanjing University Medical School, The First People's Hospital of Yancheng Yancheng Jiangsu China; ^3^ Yancheng Engineering Technology Research Center Yancheng Jiangsu China; ^4^ The Fourth People's Hospital of Yancheng Yancheng Jiangsu China; ^5^ Nanyang Town Central Hospital Yancheng Jiangsu China; ^6^ Jiangsu Key Laboratory of New Drug Research and Clinical Pharmacy Xuzhou Medical University Xuzhou Jiangsu China

**Keywords:** Alzheimer's disease, myricetin, RORγt, Th17, virtual screening

## Abstract

**Aims:**

This study aimed to investigate the therapeutic potential of myricetin, a natural flavonoid, in Alzheimer's disease (AD) by targeting Th17 cell‐mediated neuroinflammation through inhibition of the transcription factor RORγt.

**Methods:**

Virtual screening of 47,963 compounds identified myricetin as a potential RORγt inhibitor, validated by molecular docking and dynamics simulations. In vivo, 3xTg‐AD mice were treated with myricetin (50 mg/kg/day) for 8.5 weeks, followed by behavioral tests (novel object recognition, Morris water maze) and pathological analyses (HE/Nissl staining, immunohistochemistry, Western blot). In vitro, Th17 polarization assays and mechanistic studies (ChIP, EMSA, MST) were performed to elucidate myricetin's action on RORγt‐IL‐17 signaling.

**Results:**

Myricetin exhibited stable binding to RORγt (Kd = 6.40 μM), suppressing Th17 polarization and IL‐17 production. In AD mice, myricetin improved cognitive function, reduced neuronal damage, decreased Aβ plaques and phosphorylated tau levels, and attenuated microglial activation. Mechanistically, myricetin blocked RORγt recruitment to the *IL17* promoter, downregulating IL‐17 transcription.

**Conclusion:**

Myricetin ameliorates AD pathology by inhibiting RORγt‐driven Th17 polarization, highlighting its potential as a therapeutic agent for AD.

## Graphical abstract

Myricetin inhibits RORγt‐driven Th17 polarization, reducing neuroinflammation and ameliorating cognitive deficits and pathological features in Alzheimer's disease mice, highlighting its potential as a therapeutic agent for AD.

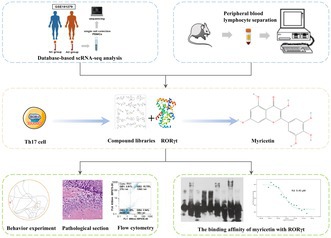

## 1 Introduction

Alzheimer's disease (AD) is the most common neurodegenerative disease among the elderly worldwide, clinically manifested as memory decline, cognitive impairment, emotional and personality changes, etc. [1]. It is estimated that by 2030, the total social cost of AD may exceed 3 trillion yuan [2]. The primary pathological features of AD include extracellular Aβ deposition and intracellular neurofibrillary tangles (NFTs) [3]. Despite decades of scientific efforts, the treatment of AD remains a significant challenge, with current therapies offering only mild symptomatic relief and limited duration of efficacy [4].

Recent research has highlighted the critical role of T helper 17 (Th17) cells in the pathogenesis of AD. Th17 cells are known for secreting pro‐inflammatory cytokines, which are associated with various neurological disorders, including AD [5]. In AD model rats, Th17 cells can cross the damaged blood–brain barrier (BBB), infiltrate the brain parenchyma, and directly induce neuronal apoptosis through the release of cytokines and the Fas/FasL pathway, leading to neuroinflammation and neurodegenerative changes [6]. Microarray analysis of peripheral blood mononuclear cells from AD patients has shown upregulation of molecules related to Th17 cells, supporting the presence of an autoimmune component in AD pathogenesis [7]. Additionally, a case–control study found that the number of circulating Th17 cells increases in the early stages of AD, while regulatory T cells (Tregs) are correlated with tau protein‐related neurodegenerative markers [8].

RORγt is a key transcription factor for the development and function of Th17 cells and plays a crucial role in inflammatory autoimmune diseases [9]. RORγt regulates the expression of IL‐17 and IL‐17F in CD4^+^ T cells and is essential for Th17 cell polarization in response to IL‐6 and TGF‐β [10]. The expression of RORγt is regulated by multiple signaling pathways and epigenetic mechanisms [11]. Given its central role in Th17 cell development and autoimmune responses, RORγt is considered a potential therapeutic target for inflammatory autoimmune diseases [12]. In the pathological process of AD, neuroinflammation is a significant feature, with the activation of microglia and the release of pro‐inflammatory cytokines being associated with disease progression [13]. Furthermore, RORγt‐mediated signaling pathways may be involved in neuronal survival, synaptic plasticity, and the deposition and clearance of Aβ protein [14].

In the critical path of drug development, the discovery and optimization of lead compounds are core steps that determine the success of new drug development, and their quality directly affects the direction and efficiency of subsequent preclinical research [15]. With the rapid development of computer science and technology, virtual screening has become a cutting‐edge technique in the field of drug discovery [16]. Virtual screening can accurately identify potentially active compounds from vast and complex chemical libraries for specific targets, opening a new path for lead compound discovery [17]. This study aims to identify potential RORγt inhibitors by combining virtual screening, drug informatics analysis, and molecular dynamics simulations. Through systematic analysis of many candidate compounds using computer‐aided virtual screening technology, this study has identified the natural flavonoid myricetin as a potential RORγt inhibitor for the first time.

## 2 Materials and Methods

### 2.1 Single‐Cell Sequencing Data Acquisition and Processing

We downloaded the GSE181279 data set based on the GPL24676 platform (Illumina NovaSeq 6000, *Homo sapiens* ) from the Gene Expression Omnibus (GEO) database and processed the single‐cell RNA sequencing data using the Seurat R package (version 4.3.0).

### 2.2 Ligand Library and Protein Receptor Preparation

We extracted 47,963 compounds from the ZINC15 database (Sterling and Irwin, J. Chem. Inf. Model, 2015). The three‐dimensional structure file of RORγt was obtained from the RCSB Protein Data Bank, and the protein receptor was prepared using Schrödinger software by adding hydrogen atoms and removing water molecules.

### 2.3 Virtual Screening and Molecular Docking

The RORγt protein was screened against the 47,963 compounds using the Glide high‐throughput virtual screening (HTVS) module in Schrödinger software. The screening results were ranked based on Glide Scores (G Scores), and standard precision (SP) and extra precision (XP) docking modes were used to improve screening accuracy.

### 2.4 Molecular Dynamics Simulation

Molecular dynamics simulations were performed on myricetin to further understand its stability under biological conditions. The Desmond system builder was used to establish a TIP3P solvent model. The Desmond molecular dynamics module was set to run for 100 ns, generating approximately 1000 frames.

### 2.5 Animal Model Preparation and Experimental Protocol

The triple‐transgenic (3xTg) AD mouse, B6‐Tg (APPSwe, tauP301L) PS1tm1 (APP/PS1/tau), aged 8–10 weeks, was purchased from HFK Bioscience Co. Ltd. (Beijing; license number SYXK 2018‐0008). Wild‐type (WT) mice were used as a blank control. Only female 3xTg mice were included in this study due to the large neuropathological variability of male mice [18, 19, 20, 21]. Myricetin (M813619, Macklin Co.) was dissolved in 0.5% (w/v) sodium carboxymethyl cellulose and administered via gavage at a dose of 50 mg/kg/day into 8‐month‐old mice for 8 weeks. All procedures complied with the experimental animal use management standards and ethical guidelines established by the Jiangsu Medical College Animal Ethics Committee.

### 2.6 In Vitro Polarization of Th17 Cells

CD4^+^ naïve T cells were purified using a magnetic cell sorting kit according to the protocol (130–104‐453, Miltenyi). For Th17 polarization, the purified CD4^+^ naïve T cells were cultured for 3 days in RPMI‐1640 containing 10% FBS, 1 mM glutamine, 0.1 mM β‐mercaptoethanol, 1% non‐essential amino acids, anti‐CD3 and anti‐CD28‐coated beads (11452D, Thermo Fisher Scientific Inc.), 5 ng/mL IL‐2, 20 ng/mL IL‐6, 5 ng/mL TGF‐β, 10 ng/mL IL‐23, 2 μg/mL anti‐IL‐4, and 2 μg/mL anti‐interferon‐γ (BD Bioscience). In some experiments, 100 μM myricetin was added to the medium and cultured for 24 h.

### 2.7 Novel Object Recognition (NOR)

At the start of the experiment, two identical cylindrical objects were placed in the open field. After 24 h, one of the cylindrical objects was replaced with a new rectangular object. The movement trajectories and interaction times with the familiar and novel objects were recorded using Any‐maze software. The novel object recognition index (%) was calculated as follows: (Time exploring novel object/(Time with novel object + Time with familiar object)) × 100%.

### 2.8 Morris Water Maze

On the first day of the training phase, the platform was raised 1 cm above the water surface to ensure visibility. From the second to the fifth day, the platform was submerged 1 cm below the water surface. Mice were placed at marked starting points in each quadrant and allowed to navigate freely in the water maze for 60 s. On the fifth day, the platform was removed. Mice were placed in the pool and allowed to move freely for 60 s. The AnyMaze software recorded and analyzed the activity time and movement trajectories of the mice in each quadrant.

### 2.9 Hematoxylin and Eosin (HE) Staining

Paraffin‐embedded tissue sections were sequentially dewaxed in xylene and washed through a series of graded ethanol solutions. Then, the sections were stained with hematoxylin and eosin solution. Microscopic images of neuronal morphology were captured using a microscope (Olympus). The Image J software was used to quantify the damaged neurons.

### 2.10 Nissl Staining

Paraffin sections were stained with toluidine blue for 1–2 min and dried in an oven at 65°C. The Olympus SlideView VS200 slide scanner was used for panoramic scanning of the sections. The Image J software was used to quantify the damaged neurons.

### 2.11 Thioflavin T Staining

Brain tissue sections were first treated with a thioflavin T solution for 5 min and immersed in 70% ethanol for 5 min to further fix the staining. Then, the brain sections were mounted on slides for observation under a fluorescence microscope, and corresponding image data were collected.

### 2.12 Histological Immunostaining

Paraffin‐embedded tissue sections were first dewaxed and hydrated. Next, primary antibodies against Iba‐1 (019‐19741, WAKO), Aβ (ab201061, Abcam), GFAP (ab207165, Abcam), APP (255241‐AP, Proteintech), P‐Tau (28666‐1‐AP, Proteintech), RORγt (bs‐10647R, Bioss), or IL‐17A (bs‐2140R, Bioss) were applied and incubated overnight at 4°C. The following day, a horseradish peroxidase (HRP) labeled secondary antibody was applied and incubated at 37°C; then DAB chromogen or tyramide signal amplification (TSA) fluorescent dye was used for staining. Microscopic images were captured, and the positively stained areas in each micrograph were analyzed and quantified using ImageJ software.

### 2.13 Western Blot (WB)

Hippocampal and cortical tissues were homogenized to collect the protein‐containing supernatant for electrophoresis. After protein transfer, the membranes were blocked for 1 h, washed, and incubated with primary antibodies: APP (255241‐AP, Proteintech), P‐Tau (28666‐1‐AP, Proteintech), GAPDH (AF2819, Beyotime) overnight at 4°C. The following day, after washing, secondary antibodies were applied and incubated for 1 h, followed by further washing and the addition of developing solution for visualization, thereby completing the Western blot analysis.

### 2.14 Quantitative RT‐PCR

RNA was extracted from cell lysates using TRIzol reagent (Invitrogen) and reverse transcribed into cDNA using the QuantScript RT kit (KR103, Tiangen). The resulting cDNA was further analyzed using the SYBR Green real‐time PCR kit (04707516001, Roche) on the LightCycler 480 system (Roche). The expression levels were determined using the 2^−ΔΔCt^ method. The primers used were as follows: RORγt, Forward: 5′‐TGCAAGACTCATCGACAAGG‐3′, Reverse: 5′‐AGGGGATTCAACATCAGTGC‐3′; IL‐17, Forward: 5′‐TTTAACTCCCTTGGCGCAAAA‐3′, Reverse: 5′‐CTTTCCCTCCGCATTGACAC‐3′; β‐actin, Forward: 5′‐GAGACCTTCAACACCCCAGCC‐3′, Reverse: 5′‐AATGTCACGCACGATTTCCC‐3′.

### 2.15 Flow Cytometry

Lymphocytes were isolated from peripheral blood samples of each group of mice using Percoll gradient centrifugation (LTS1092, TBD). After stimulation, the cells were stained with CD4 for 30 min. Next, the cells were fixed, followed by staining with IL‐17A (17‐7177‐81, Invitrogen) and RORγt (ab104906, Abcam) for 45 min. After incubation, the cells were transferred to flow cytometry tubes for analysis using a flow cytometer (Sysmex).

### 2.16 Enzyme‐Linked Immunosorbent Assay (ELISA)

The concentrations of IL‐17A in the supernatants of cultured cells and soluble Aβ in tissue were determined using ELISA kits (Cat No. JL20251, JL11386, Jonln bio.), strictly adhering to the protocols provided by the manufacturer.

### 2.17 *il17* Promoter Sequence Analyses

The *il17* promoter sequence was obtained from UCSC (http://genome.ucsc.edu/ENCODE/). RORγt binding sites were predicted using the JASPAR open access database (jaspar.genereg.net/) following website instructions.

### 2.18 Chromatin Immunoprecipitation (ChIP) Assay

ChIP was performed using an EZ‐ChIP kit (17‐371, Sigma‐Aldrich). The bound chromatin was eluted for quantitative PCR tests. Primers for amplifying fragments covering the RORγt binding site were listed as follows: Forward: 5′‐ATGATGGGAACTTGAGTA‐3′, Reverse: 5′‐GCTGCTATGCTATGGGTC‐3′.

### 2.19 Dual‐Luciferase Reporter Assay

The *il17* promoter sequence was ligated into the pGL4.20[luc2Puro] vector. Recombinant plasmids and the internal control vector PRL‐TK Renilla vector were transfected into cells using Lipofectamine 3000 reagent (Invitrogen). After 48 h, cells were harvested and measured for luciferase activity using the Dual‐Luciferase Reporter Assay System (E1910, Promega).

### 2.20 Electrophoretic Mobility Shift Assay (EMSA)

The cellular nuclear protein was extracted, and then mixed with 0.1 μM Biotin‐labeled double‐stranded oligonucleotides. As a control, extra unlabeled competitor oligonucleotide or mutant competitor oligonucleotide was used. RORγt antibody (bs‐10647R, Bioss) was needed in the super‐shift reaction. Mixtures were analyzed by electrophoresis in 4% polyacrylamide gels, and then transferred to a nylon membrane for chemiluminescence with HRP‐conjugated Streptavidin. The probes (Sangon) were listed as follows: Probe, 5′‐AGGTACATGACACCAGA*AGACCTACATGT*TACTTCAAACT‐Biotin; Specific competitor, 5′‐AGGTACATGACACCAGA*AGACCTACATGT*TACTTCAAACT‐3′; Mutant competitor, 5′‐AGGTACATGACACCAGA*CTCAAGCACGTG*TACTTCAAACT‐3′. Italic characters represented binding motifs or mutation sequences.

### 2.21 Microscale Thermophoresis Assay

The binding affinity of myricetin with RORγt was analyzed by microscale thermophoresis (MST). Purified RORγt (CSB‐MP020071MO, CUSABIO) was labeled with the Monolith NT Protein Labeling Kit RED (NanoTemper Technologies). Serially diluted myricetin, with concentrations of 2.4 mM to 73.24 nM, was mixed with 80 nM labeled RORγt at room temperature and loaded into Monolith TM standard‐treated capillaries. Binding was measured by monitoring the thermophoresis on a Monolith NT.115 instrument (Nano Temper Technologies).

### 2.22 Statistical Analysis

Data analysis was performed using IBM SPSS Statistics 26.0, and graphs were generated using GraphPad Prism 6.0. Quantitative results are presented as mean ± SEM. Comparisons between two groups were performed using t‐tests, while comparisons among multiple groups were conducted using ANOVA followed by Bonferroni's post hoc test. A *p*‐value ≤ 0.05 was considered statistically significant.

## 3 Results

### 3.1 Increased Proportion of Th17 Cells in Peripheral Blood Mononuclear Cells From AD Patients

**FIGURE 1:**
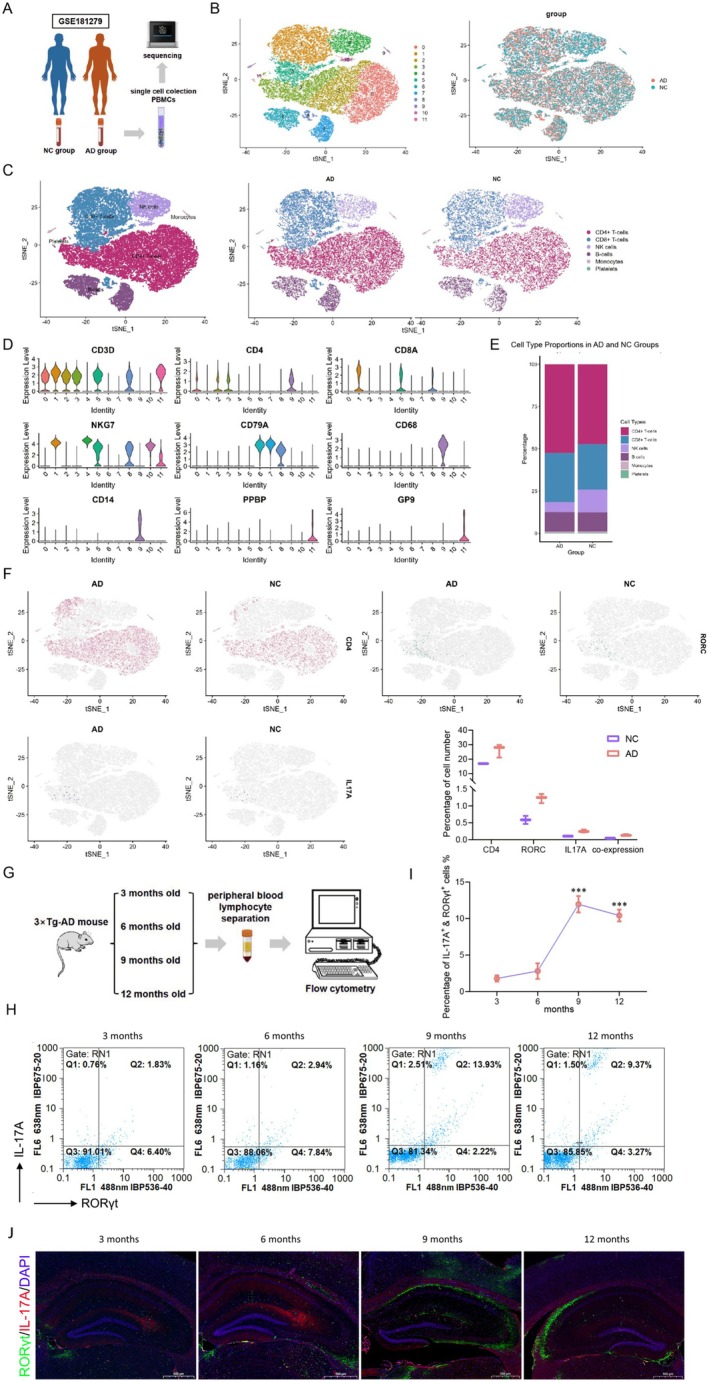
Increased proportion of Th17 cells in AD patients and mice. (A) Flowchart of data acquisition from the single‐cell sequencing data set GSE181279 in the GEO database. (B) t‐SNE plot of 34,713 cells from the AD group (*n* = 21,224) and NC group (*n* = 13,489). (C) t‐SNE plot after classification of all cells. (D) Violin plots showing the expression levels of typical cell marker genes in each cluster. (E) Bar graph comparing the proportions of different cell types between the AD and NC groups. (F) Expression levels and proportions of CD4, RORγt, and IL17A in all cells. (G) Flowchart of Th17 cell proportion detection in peripheral blood mononuclear cells from AD mice at different ages. (H, I) Tests of Th17 cell proportions in peripheral blood mononuclear cells from AD mice at different ages. (J) Immunofluorescence images of RORγt and IL‐17A in the hippocampus of AD mice at different ages. Bars, 500 μm. ***p < 0.001.

We downloaded the GSE181279 dataset based on the GPL24676 platform (Illumina NovaSeq 6000, *Homo sapiens* ) from the GEO database (Figure 1A) [22]. After quality control processing of the sequencing data, we obtained 34,713 high‐quality cells from AD patients and negative controls (NC). After normalization, batch effect removal, and dimensionality reduction, t‐SNE was used to visualize 12 distinct cell clusters and the distribution of cells from the AD and NC groups (Figure 1B). Following cell annotation, five major immune cell types were identified (Figure 1C,D). As shown in Figure 1E, the proportion of CD4^+^ T cells was higher in the AD group than in the NC group. In addition, the proportions of cells expressing Th17 specific genes CD4, RORγt, IL17A individually or co‐expressing all three genes were higher in the AD group than in the NC group (Figure 1F).

To further determine the correlation between Th17 disorder and AD progression, peripheral blood lymphocytes were isolated from 3 × Tg AD mice at 3, 6, 9, and 12 months of age, and the proportions of IL17A^+^ and RORγt^+^ cells were detected using flow cytometry (Figure 1G,H). The results indicated that the proportion of Th17 cells in the peripheral blood of 9‐month‐old AD mice reached a peak compared with that in 3‐month‐old, 6‐month‐old, and even 12‐month‐old AD mice (Figure 1I). Consistently, more RORγt^+^ Th17 cells were observed in 9‐month‐old AD mice (Figure 1J).

In summary, the increased proportion of Th17 cells in the peripheral blood mononuclear cells of both AD patients and mice suggests that Th17 cells may be a contributing factor in the progression of AD.

### 3.2 Myricetin Is Screened to Exhibit Stable Binding and Strong Affinity With RORγt

**FIGURE 2:**
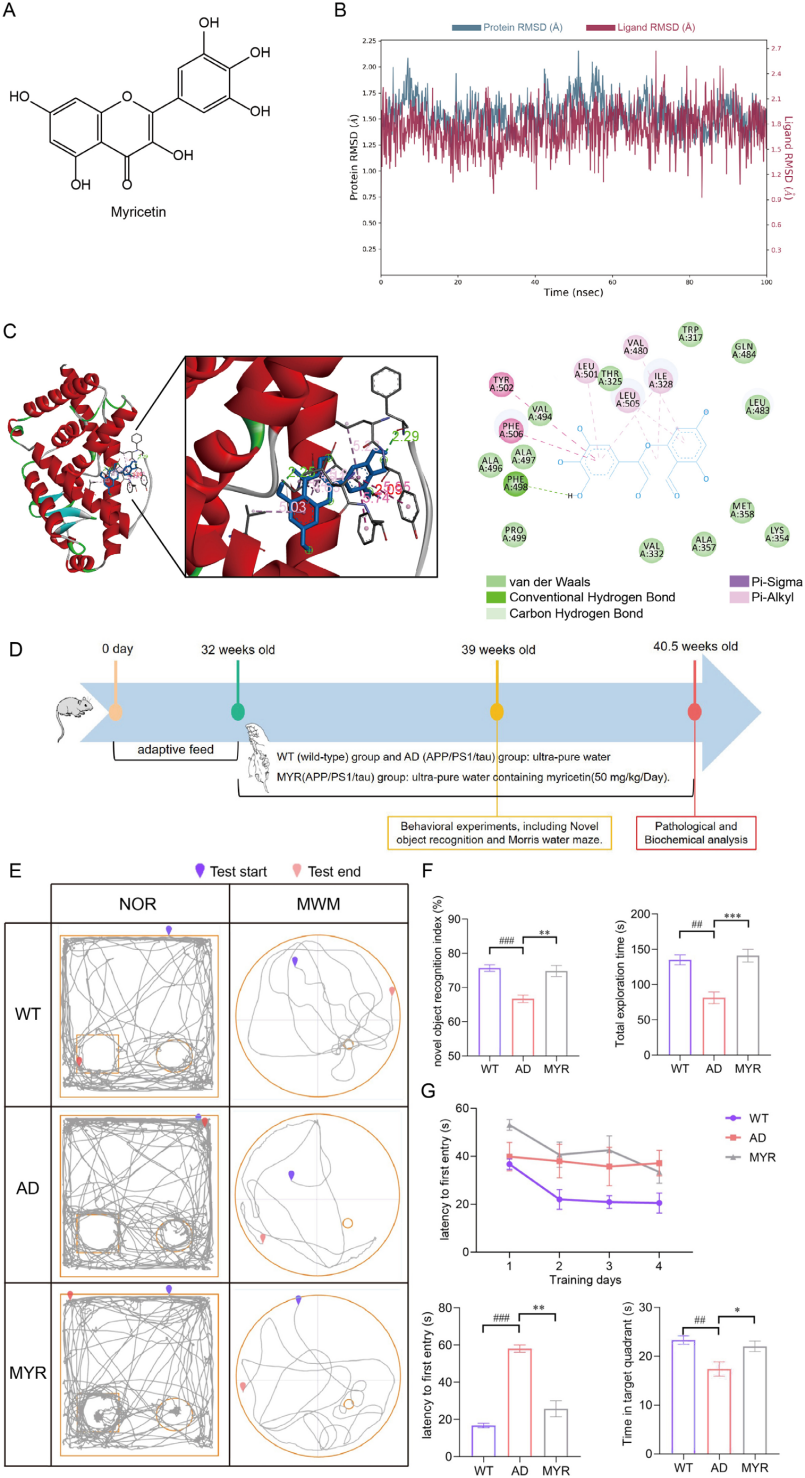
Molecular dynamics and effects of myricetin on behavioral performance in AD mice. (A) Molecular structure of myricetin. (B) Dynamic changes in the RMSD of myricetin and RORγt over time. (C) 3D and 2D molecular docking diagrams of myricetin and RORγt. (D) Experimental workflow for animal studies. (E) Movement trajectories of mice in the novel object recognition and Morris water maze tests (*n* = 10). (F) Novel object recognition index and total exploration time (*n* = 10). (G) Latency to first entry and time spent in the target quadrant (*n* = 10). ^##^ *p* < 0.01, ^###^ *p* < 0.001 compared to the WT group; *p < 0.05, **p < 0.01, ***p < 0.001 compared to the AD group.

After high‐throughput virtual screening, myricetin (Figure 2A), which had the highest G Score, was selected for molecular dynamics simulations with RORγt. The root mean square deviation (RMSD) values of the RORγt protein chain‐myricetin complex remained below 3, and the overall fluctuation trend demonstrated good stability, indicating that the binding between RORγt and myricetin is stable (Figure 2B). To further investigate the binding strength between RORγt and myricetin at the molecular level, we conducted molecular docking experiments. The results revealed significant interactions between RORγt and myricetin, with the formation of multiple hydrogen bonds and carbon‐hydrogen bonds (Figure 2C). In addition, the calculated binding energy is −7.42 kcal/mol, and this value indicates that there is a strong binding affinity between them.

### 3.3 Myricetin Improves Learning and Memory Impairments in AD Mice

To investigate the effects of myricetin on learning and memory abilities in AD mice, myricetin was administered daily to 8‐month‐old 3xTg mice via gavage. Behavioral experiments were conducted to assess learning and memory abilities; then, the 10‐month‐old mice were anesthetized and euthanized to collect tissue samples for subsequent pathological and molecular biological analyses (Figure 2D).

In the NOR test, the recognition index of AD mice was significantly lower than that of WT mice, while this index in the myricetin group was significantly higher compared with the AD group (Figure 2E,F). The results of the MWM test showed that compared to the WT group, AD mice exhibited significantly longer latencies to first reach the platform during the 4‐day training and the test on the fifth day, with significantly shorter time spent in the target quadrant and fewer platform crossings. Compared with AD mice, myricetin‐treated mice showed significantly shorter latencies to first reach the platform, longer time spent in the target quadrant, and increased platform crossings (Figure 2E,G). These results indicate that myricetin can significantly improve the spatial learning and memory impairments of AD mice.

### 3.4 Myricetin Ameliorates Pathology in AD Mice

**FIGURE 3:**
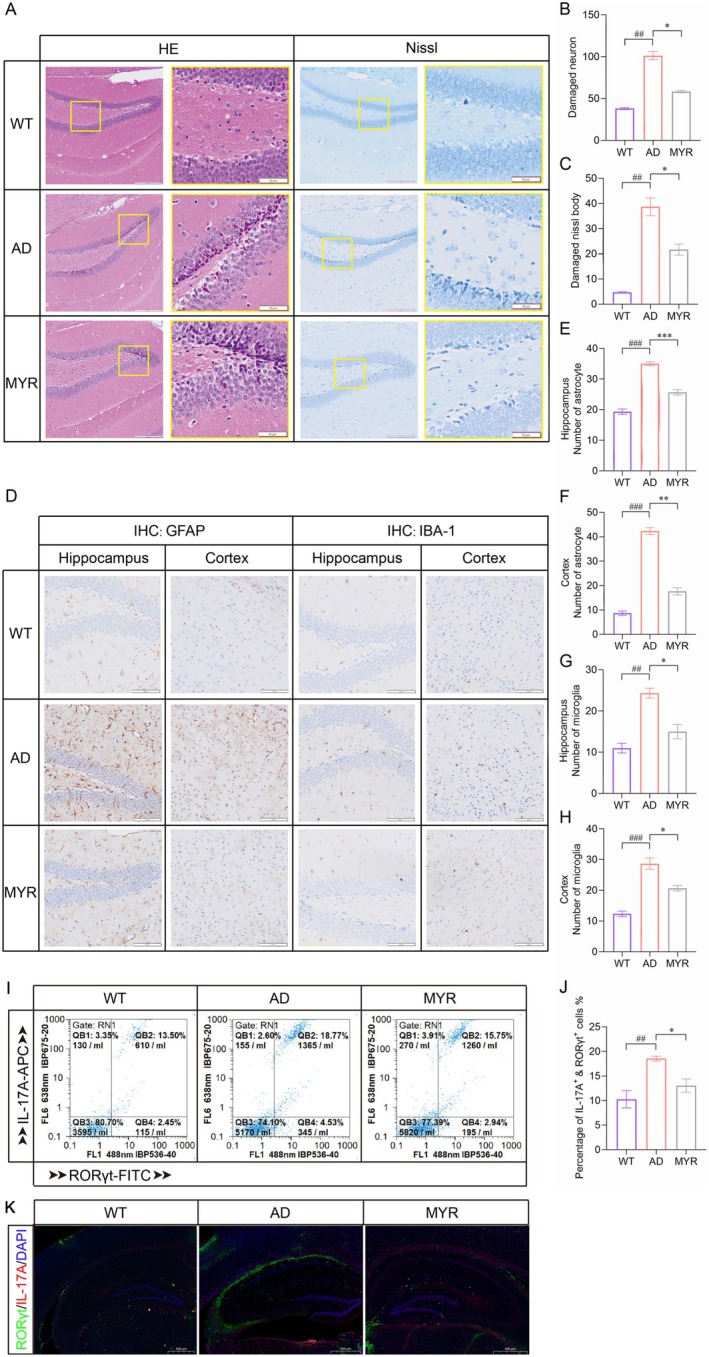
Neuroprotective and immunomodulatory effects of myricetin in AD mice. (A) HE staining and Nissl staining of the hippocampus (*n* = 5). Bars, 200 μm for low magnification and 50 μm for high magnification. (B) Number of damaged neurons. (C) Damaged Nissl bodies. (D) Immunohistochemical images of GFAP and IBA‐1 in the hippocampus and cortex (*n* = 5). Bars, 100 μm. (E) Number of astrocytes in the hippocampus. (F) Number of astrocytes in the cortex. (G) Number of microglia in the hippocampus. (H) Number of microglia in the cortex. (I, J) Proportions of IL‐17A^+^ RORγt^+^ cells in peripheral blood lymphocytes (*n* = 5). (K) Immunofluorescence images of RORγt and IL‐17A in the hippocampus (*n* = 5). Bars, 500 μm. ^##^ *p* < 0.01, ^###^ *p* < 0.001 compared to the WT group; *p < 0.05, **p < 0.01, ***p < 0.001 compared to the AD group.

To investigate the role of myricetin in ameliorating AD pathology, HE staining results revealed that the hippocampal tissue in the AD group exhibited a disorganized arrangement and increased neuronal damage, while the myricetin treated mice showed reduced neuronal damage, with decreased nuclear pyknosis and neuronal loss compared to the AD group (Figure 3A,B). Similarly, Nissl staining of hippocampal neurons in mice demonstrated that the hippocampal neurons in WT mice had intact structures and clear nucleoli, while the hippocampal region of AD model mice showed a significant increase in nuclear pyknosis and damaged neurons, which were decreased after myricetin treatment (Figure 3A,C).

IHC analysis of Iba‐1 and GFAP was performed to quantify the numbers of microglia and astrocytes in the hippocampus and cortex of the brain. Compared to WT mice, the number of astrocytes and activated microglia in the hippocampus and cortex of AD model mice was significantly increased, while it was significantly reduced after myricetin treatment (Figure 3D–H). In addition, myricetin‐treated AD mice exhibited a significantly decreased proportion of RORγt^+^ Th17 cells both in the peripheral blood lymphocytes (Figure 3I,J) and hippocampus (Figure 3K), approaching the normal physiological level in WT mice (Figure 3I–K).

**FIGURE 4:**
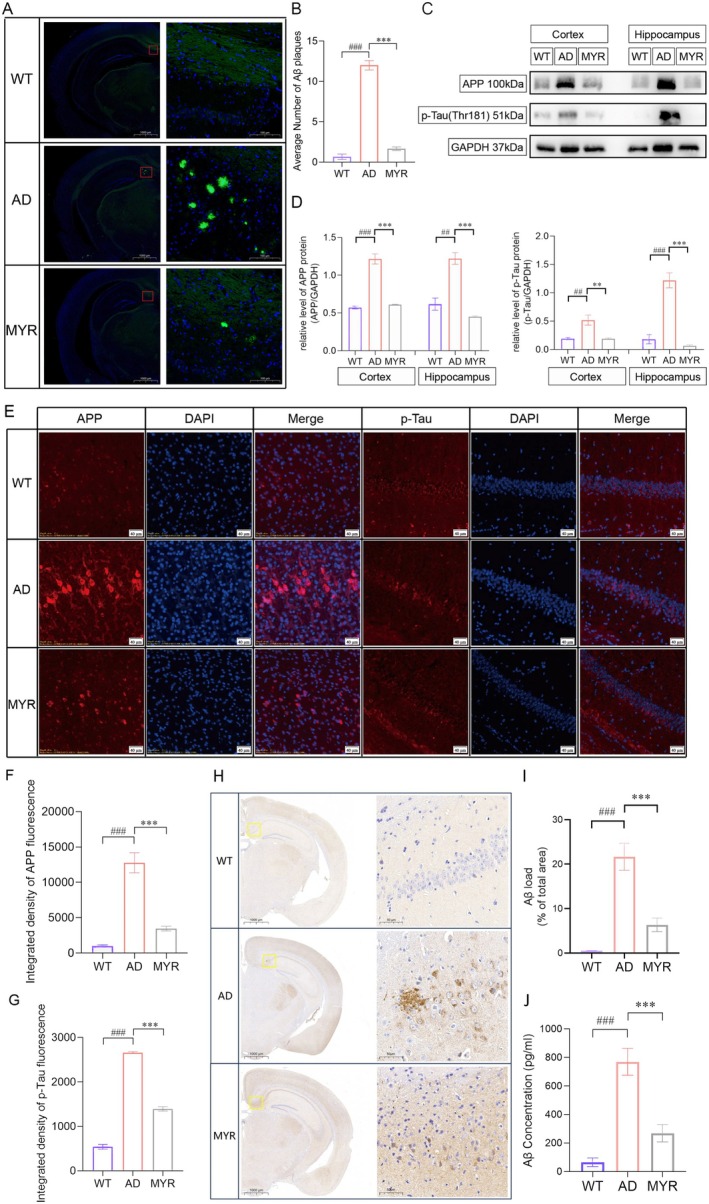
Improving effects of myricetin on AD pathology. (A) Thioflavin T staining in the hippocampus (*n* = 5). Bars, 1000 μm for low magnification and 100 μm for high magnification. (B) Number of Aβ‐positive plaques. (C) Representative Western blot bands of APP, P‐Tau, and GAPDH (*n* = 5). (D) Relative expression levels of APP and P‐Tau proteins. (E) Fluorescence images of APP and P‐Tau (*n* = 5). Bars, 40 μm. (F, G) Analysis of APP and P‐Tau fluorescence intensity. (H, I) Immunohistochemical images and analysis of Aβ in mice (*n* = 5). Bars, 1000 μm for low magnification and 50 μm for high magnification. (J) ELISA of Aβ concentration in serum (*n* = 5). ^##^ *p* < 0.01, ^###^ *p* < 0.001 compared to the WT group; **p < 0.01, ***p < 0.001 compared to the AD group.

We further investigated the effects of myricetin on the Aβ plaques as well as the expression levels of APP and phosphorylated tau protein (P‐Tau) in mice. Compared to AD mice, significantly fewer TS^+^ Aβ plaques were seen after myricetin treatment (Figure 4A,B). Western blot analysis revealed that AD mice exhibited a significant upregulation of APP and P‐Tau expression in the cortex and hippocampus, while these expression levels were significantly decreased in the myricetin treated AD mice (Figure 4C,D). Consistently, myricetin treatment significantly reduced the fluorescence intensity of APP and P‐Tau in AD mice (Figure 4E–G). Moreover, compared to WT mice, the amount of Aβ in the cortex and serum of AD model mice was significantly increased, while it was significantly reduced after treatment with myricetin (Figure 4H–J). These data demonstrate that myricetin ameliorates pathology and inflammation in AD mice.

### 3.5 Myricetin Suppresses Th17 Polarization via Blocking the Transcriptional Activity of RORγt

**FIGURE 5:**
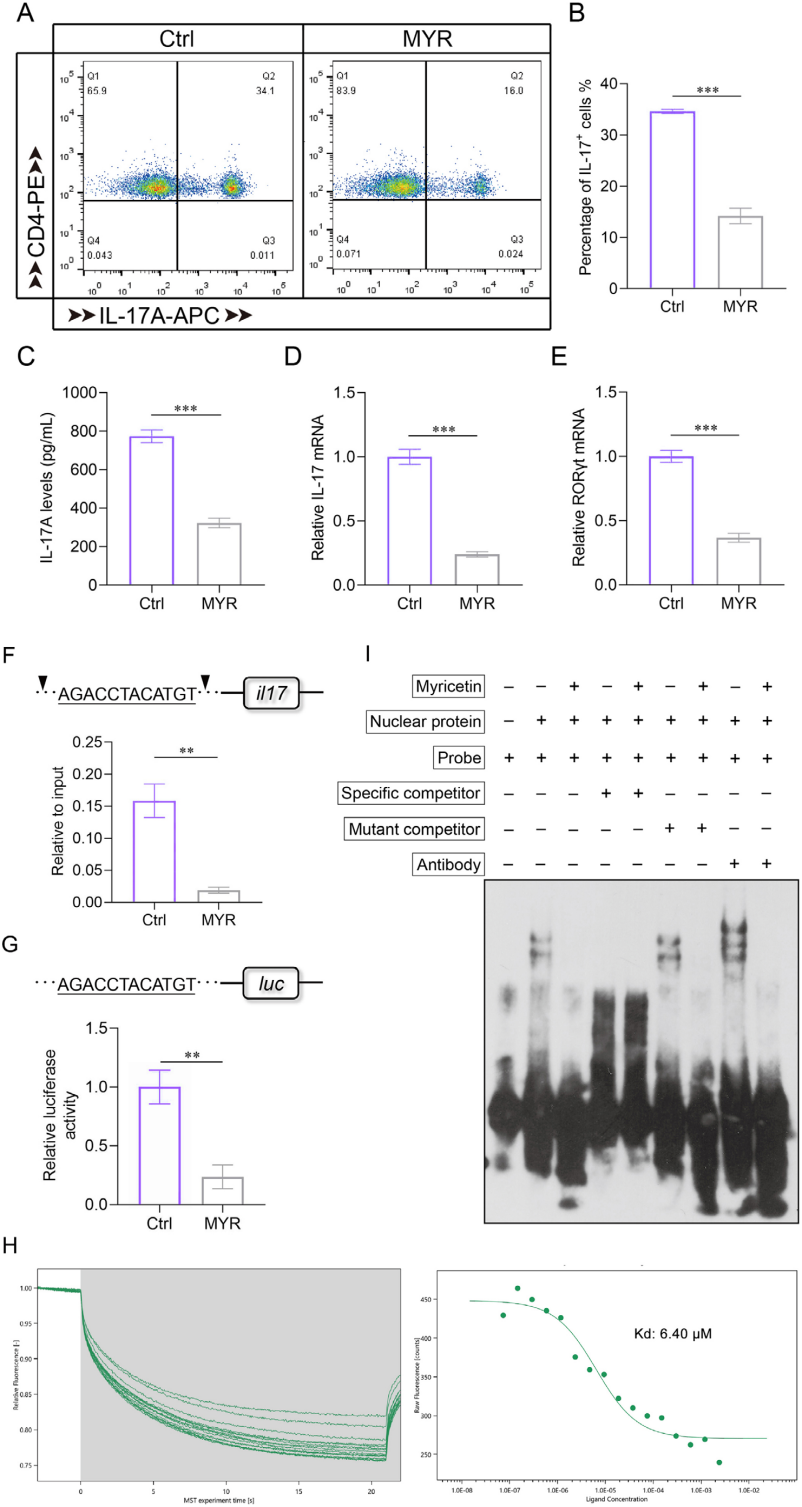
Binding of myricetin with RORγt blocks Th17 polarization in vitro. (A, B) Proportions of CD4^+^IL‐17A^+^ cells. (C) Concentration of IL‐17A in the culture supernatant. (D, E) Levels of IL‐17A and RORγt mRNA. (F) Recruitment levels of RORγt at target site located in the *il17* promoter. Black arrows indicate primer binding sites in the *il17* promoter. The values were normalized to the input for each sample. (G) Luciferase activity test by dual‐luciferase reporter assay. The values were normalized to Renilla luciferase activity. (H) Determination of binding affinity between myricetin and RORγt by MST. (I) Combination test between RORγt and target sequence by EMSA. **p < 0.01, ***p < 0.001 compared to the Ctrl group.

To explore the role and mechanism of myricetin on Th17 polarization, we added myricetin into medium during the polarization of Th17 in vitro. The results showed that myricetin significantly decreased the proportion of IL‐17A‐positive cells (Figure 5A,B), the secretion of IL‐17A (Figure 5C), and the expression of IL‐17 and RORγt (Figure 5D,E), suggesting an inhibitory effect on Th17 polarization in vitro.

To further investigate the reasons why myricetin inhibits the expression of IL‐17, we scanned the *IL‐17* promoter sequence and identified a RORγt binding site (Figure 5F). By ChIP test, the recruitment amount of RORγt near the binding site was sharply decreased after myricetin treatment (Figure 5F). Moreover, the result of the dual‐luciferase reporter assay showed that myricetin could lead to a much lower transcriptional activity than that of the control (Figure 5G).

Next, the direct binding of myricetin with RORγt was verified by MST and EMSA. The MST binding assay revealed that myricetin bound to RORγt at a low Kd value of 6.40 μM (Figure 5H). Moreover, the result of EMSA showed that RORγt bound to the target probe and this interaction disappeared by competition with a specific unlabeled probe, while the mutant competitive probe had no effect (Figure 5I, lanes 2, 4, 6). Moreover, a super‐shifted band further indicated the binding of RORγt and the target probe (Figure 5I, lane 8). However, after myricetin treatment, all the combination bands disappeared (Figure 5I, lanes 3, 7, 9), suggesting a blocking effect of myricetin on the binding of RORγt with the target probe. Taken together, these data demonstrate that myricetin can downregulate IL‐17 expression by directly binding with RORγt to block its anchoring on the *il17* promoter.

## 4 Discussion

In this study, through virtual screening, molecular dynamics simulations, and experimental validation, our findings demonstrate that myricetin not only significantly suppresses Th17 cell polarization by blocking RORγt transcriptional activity, but also improves cognitive dysfunction and neuronal damage in AD mice, providing new insights and a potential candidate drug for AD treatment.

We observed an increased proportion of Th17 cells in the peripheral blood mononuclear cells (PBMCs) of both AD patients and AD mouse models, which aligns with previous studies highlighting the critical role of Th17 cells in AD pathogenesis [23]. Th17 cells disrupt the BBB and, induced by Aβ1‐42, elevate pro‐inflammatory cytokines, interleukin (IL)‐17, and IL‐22 in the AD brain [24]. A study on 3xTg‐AD mice found that treatment with an IL‐17 monoclonal antibody prevented short‐term cognitive deficits [25]. RORγt is a key transcription factor for Th17 cell polarization, initiating and promoting their polarization while maintaining their functional characteristics and regulating the expression of specific cytokines such as IL‐17A, IL‐17F, and IL‐22 [26]. Thus, we employed virtual screening technology [27] to screen potential RORγt inhibitors from 47,963 compounds in the ZINC15 database, significantly narrowing the research scope. Based on the screening results, we selected myricetin as the focus of our study.

Myricetin, a natural product with multiple biological activities, has been shown in numerous studies to possess antioxidant and anti‐inflammatory properties [28]. At the cellular level, myricetin significantly inhibits hypoxia‐induced activation of microglia toward the M1 phenotype, primarily through the regulation of the STAT1 signaling pathway, while also demonstrating significant neuroprotective effects that effectively prevent neuronal death [29]. More importantly, in the field of AD research, myricetin has shown multifaceted therapeutic potential: it not only alleviates Fe^2+^‐induced SH‐SY5Y cell death but also inhibits acetylcholinesterase (AChE), reduces brain iron content, reverses scopolamine‐induced cognitive dysfunction, and mitigates oxidative stress damage in AD mouse models [30]. Researchers have found that myricetin increases the number of CA3 pyramidal neurons in the hippocampus and ameliorates learning and memory impairments in AD rats through behavioral tests and tissue staining [31]. Histopathological staining and immunofluorescence detection results further confirmed the ameliorative effects of myricetin on AD pathological features. Similarly, studies have found that myricetin, through its antioxidant effects on cell membranes and mitochondria, effectively prevents neurotoxicity and mitochondrial dysfunction induced by high‐molecular‐weight Aβ1‐42 oligomers, demonstrating therapeutic potential for AD [32]. This suggests that myricetin may delay AD progression through multiple pathways, not only by inhibiting Th17 cell‐mediated neuroinflammation but also by influencing Aβ metabolism and tau protein phosphorylation.

As AD progresses, the number of Th17 cells in the peripheral blood of mice showed an initial increase followed by a decline. The early increase may be primarily related to changes in the cytokine environment: elevated IL‐1β and reduced IL‐2 levels drive the expansion of Th17 cells [33]. Additionally, the persistently activated immune system due to immune senescence, along with changes in the microbial community, further facilitates Th17 cell proliferation [34, 35]. Thus, the increased proportion of Th17 cells in the peripheral blood may be one of the key causes of AD onset. However, with the further development of AD, Th17 cells may no longer play the leading role, and the gradually declined number of Th17 cells may be attributed to the regulation of the adaptive immune response: increased apoptosis of Th17 cells (potentially due to enhanced oxidative stress and activation of apoptotic pathways) and enhanced function of regulatory T cells, which exert stronger inhibitory effects on Th17 cells [34, 36].

Although this study specifically elucidates how myricetin ameliorates AD pathology through Th17 polarization inhibition, current research highlights myricetin's ability to orchestrate multiple immune cell subsets, potentially establishing synergistic effects against neuroinflammation while promoting neuroprotective mechanisms [37]. In experimental autoimmune myocarditis models, myricetin rebalanced Th17/Treg ratios by suppressing Th17 cell expansion and enhancing regulatory T cell populations, effectively attenuating myocardial autoimmune pathology [38]. Parallel findings in adjuvant‐induced arthritis mice revealed that myricetin disrupted splenic follicular helper T (Tfh) cell differentiation and IL‐21 production through JAK/STAT‐Bcl‐6 axis inhibition, correlating with improved arthritic phenotypes [39]. Notably, in primary human B cells, myricetin attenuated plasma cell differentiation and immunoglobulin secretion via transcriptional reprogramming for upregulating BACH2 while suppressing IRF4/BLIMP1/XBP1 triad [40]. Beyond lymphoid lineage modulation, myricetin demonstrates myeloid cell regulatory prowess. When counteracting methylglyoxal‐induced macrophage polarization in formaldehyde‐treated tumor models, this flavonol reversed immunosuppressive microenvironment formation [41]. In diabetic nephropathy contexts, PI3K/Akt pathway‐mediated M1 macrophage polarization attenuation by myricetin significantly mitigated renal fibrosis and injury [42]. In traumatic brain injury models, myricetin dampened perilesional neuroinflammation through EGFR‐AKT/STAT‐dependent suppression of toxic microglial markers and cytokine storms [43]. AD‐specific investigations further revealed its capacity to inhibit p38 MAPK‐mediated microglial hyperactivation while inducing phenotypic switching, effectively breaking neuroinflammatory cascades [44]. Mechanistically, myricetin directly interacted with STAT1 under hypoxic conditions, disrupting S‐glutathionylation and phosphorylation processes to prevent M1 microglial activation and subsequent neuronal death [29]. Collectively, myricetin modulates immune responses by targeting diverse immune cells and suppressing key inflammatory pathways. This multi‐target action reduces pathogenic inflammation while enhancing regulatory functions, positioning it as a promising candidate for immune‐related disorders.

Numerous studies have demonstrated notable sex‐based differences in the pathological progression and molecular profiles in 3xTg‐AD mice. Specifically, female mice display more pronounced AD‐related neuropathological hallmarks compared to their male counterparts [45]. Furthermore, sex significantly influences Aβ accumulation in the hippocampus, with females exhibiting substantially greater Aβ deposition—a difference that may be linked to local estrogen concentrations [46]. Given these findings, female 3xTg‐AD mice were chosen in the present study to more effectively investigate the impact of myricetin on AD‐related pathological progression and molecular mechanisms. Future studies will include male 3xTg‐AD mice to enable a comprehensive comparison of sex‐specific responses to myricetin treatment.

## Author Contributions

Design of the project was contributed by Yufei Li, Ao Sun, and Xuebin Qu; Experimental operation and data collection were contributed by Yufei Li, Ao Sun, Rui Hong, Cong Cao, and Aihua Zhou; Data analysis and interpretation were contributed by Yufei Li, Ao Sun, Zhengxiang Fan, Linlin Zhang, Jingjing Han, and Xuebin Qu; Writing and editing were contributed by Yufei Li, Ao Sun, Jingjing Han, and Xuebin Qu.

## Ethics Statement

All experiments were performed in accordance with the Provisions and General Recommendations of the Chinese Experimental Animal Administration Legislation, as well as institutional approval from the Experimental Animal Ethics Committee of Jiangsu Medical College (the approval number: XMLL‐2022‐056).

## Conflicts of Interest

The authors declare no conflicts of interest.

## Data Availability

The data sets used and/or analyzed during the current study are available from the corresponding author on reasonable request.
